# Krüppel-Like Factor 15 Modulates *CXCL1/CXCR2* Signaling-Mediated Inflammatory Response Contributing to Angiotensin II-Induced Cardiac Remodeling

**DOI:** 10.3389/fcell.2021.644954

**Published:** 2021-04-01

**Authors:** Shun He, Yuanyuan Lu, Yuetong Guo, Shijin Li, Xiao Lu, Shuai Shao, Handan Zhou, Ruiqi Wang, Jiguang Wang, Pingjin Gao, Xiaodong Li

**Affiliations:** ^1^Department of Cardiovascular Medicine, Ruijin Hospital and State Key Laboratory of Medical Genomics, Shanghai Key Laboratory of Hypertension, Shanghai Institute of Hypertension, Shanghai Jiao Tong University School of Medicine, Shanghai, China; ^2^Department of Hypertension, Ruijin Hospital and State Key Laboratory of Medical Genomics, Shanghai Key Laboratory of Hypertension, Shanghai Institute of Hypertension, Shanghai Jiao Tong University School of Medicine, Shanghai, China

**Keywords:** hypertension, renin-angiotensin system, transcription factor, cardiac remodeling, inflammation

## Abstract

Inflammation is involved in cardiac remodeling. In response to pathological stimuli, activated cardiac fibroblasts (CFs) secreting inflammatory cytokines and chemokines play an important role in monocyte/macrophage recruitment. However, the precise mechanism of CF-mediated inflammatory response in hypertension-induced cardiac remodeling remains unclear. In the present study, we investigated the role of transcription factor Krüppel-like factor 15 (KLF15) in this process. We found that KLF15 expression decreased while chemokine *CXCL1* and its receptor *CXCR2* expression increased in the hearts of angiotensin II (Ang II)-infused mice. Compared to the wild-type mice, KLF15 knockout (KO) mice aggravated Ang II-induced cardiac hypertrophy and fibrosis. Deficiency of KLF15 promoted macrophage accumulation, increase of *CXCL1* and *CXCR2* expression, and mTOR, ERK1/2, NF-κB-p65 signaling activation in the hearts. Mechanistically, Ang II dose- dependently decreased KLF15 expression and increased *CXCL1* secretion from cardiac fibroblasts but not cardiac myoblasts. Loss- or gain-of-function studies have shown that KLF15 negatively regulated *CXCL1* expression through its transactivation domain (TAD). Intriguingly, the adenovirus-mediated full length of KLF15—but not KLF15 with TAD deletion overexpression—markedly prevented pathological change in Ang II-infused mice. Notably, the administration of *CXCR2* inhibitor SB265610 reversed KLF15 knockout-mediated aggravation of cardiac dysfunction, remodeling, and inflammation induced by Ang II. In conclusion, our study identifies that KLF15 in cardiac fibroblasts negatively regulates *CXCL1/CXCR2* axis-mediated inflammatory response and subsequent cardiac remodeling in hypertension.

## Introduction

Hypertensive heart failure is a terrible disease with high morbidity and mortality characterized by cardiac remodeling including left ventricular hypertrophy and interstitial fibrosis (Katz and Rolett, 2016). The renin-angiotensin system (RAS) plays a pivotal role in hypertension, and the sustained RAS activation contributes to the development and progression of heart failure (Te Riet et al., 2015). Clinical and experimental studies reported that deregulated Ang II, the major effector in the renin-angiotensin system, plays an important role in the pathogenesis of cardiac remodeling (Kurdi and Booz, 2011). Besides the role of cardiomyocytes and cardiac fibroblasts, other cells that reside in or infiltrate into the heart in response to Ang II also significantly participate in this process. For example, Ang II promotes endothelial cells to secrete TGF-beta, which accelerates pathological cardiac fibrosis and hypertrophy (Liu et al., 2019). Ang II also induces the infiltration of inflammatory cells, especially monocytes/macrophages in the heart and then promotes the development of heart failure (McMaster et al., 2015; Wang et al., 2018). These studies suggest that a multifaceted crosstalk between cardiac cells and non-cardiac cells contributes to cardiac remodeling. Therefore, investigation of the critical molecule that promotes these cells’ interaction in the heart after hypertensive stress may provide new therapeutic strategies for cardiac remodeling.

Krüppel-like factors (KLFs) are zinc finger-containing transcription factors involved in a broad range of functions in regulating proliferation, differentiation, development, and programmed cell death (McConnell and Yang, 2010). As a member of the KLF family, KLF15 mainly functions as a transcription repressor, the alteration of whose expression is associated with numerous diseases, including cardiovascular disease, metabolic disorders, and cancer (Yoda et al., 2015; Zhao et al., 2019). Moreover, it is reported that KLF15 is highly expressed in cardiac fibroblasts and cardiomyocytes and is essential for cardiac remodeling by controlling downstream gene expression (Noack et al., 2019). Our previous study found that Ang II induced phenotypic transformation of fibroblasts into inflammatory myofibroblasts through down-regulating KLF15 expression (Lu et al., 2019), suggesting the critical role of KLF15 in regulation of inflammatory gene expression. However, it’s important to identify the mechanism of the KLF15-mediated inflammatory response and its effects on cardiac cellular crosstalk in hypertension-induced cardiac remodeling.

Cardiac macrophages have been shown to be involved in the regulation of cardiac injury in myocardial infarction, ischemia reperfusion, and hypertension (Shirakawa et al., 2018; Wang et al., 2018; Petz et al., 2019). Chemokines and their receptors play critical roles in promoting the recruitment of monocytes/macrophages into the injured heart (Wang et al., 2018). Recently, we have found that KLF15 is necessary for repressing adventitial fibroblast-derived CCL2 excretion, which mediated macrophage infiltration into injury arteries (Lu et al., 2019). However, little is known about the function of KLF15 in cardiac fibroblasts, which regulates inflammation response-mediated cardiac remodeling in hypertension. In this study, we sought to determine whether KLF15 negatively regulates chemokine-mediated macrophage recruitment, which exacerbates cardiac hypertrophy and fibrosis in Ang II-induced hypertension.

## Materials and Methods

### Animals

All animal welfare and procedures were adhered to according to the Guide for the Care and Use of Laboratory Animals established by Shanghai Jiao Tong University School of Medicine. KLF15^flox/flox^ (C57BL/6J background) was generated by Cyagen Biosciences Inc., by introducing loxP sites flanking the coding exon 2 of the Klf15 gene. KLF15^flox/flox^ mice were crossed with CAG-Cre mice to generate global KLF15 knockout (KO) mice. The mice were maintained under the specific pathogen-free (SPF) environment with 12 h of light/dark cycles and free access to food and water. Male, 8-week-old KLF15 KO mice and WT littermates were infused by angiotensin II (1,000 ng/kg/min, Sigma, United States) for 14 days using subcutaneously implanted minipumps (Alzet, 1002) to study cardiac remodeling as previously described (Zuo et al., 2018).

Rat KLF15 (Accession no. AAH89782.1) and deletion of TAD at amino acids 132–152 (KLF15-DTAD; VSRPFQPTLEEIEEFLEENME) cloned into an adenovirus vector (padenoMCMVEGFP-P2A-3FLAG) were constructed by Obio Technology (Shanghai, China). Adenoviruses of AdCTL, AdKLF15, or AdKLF15-ΔTAD were injected through the tail vein at a dose of 1 × 10^9^ PFU per mouse 1 day before Ang II infusion. For the SB265610 treatment group, after implantation of mini-pumps infused with Ang II or saline, SB265610 (2 mg/kg/day) was intraperitoneally injected once per day for 2 weeks.

After 14 days, mice were weighed and sacrificed by intraperitoneal administration of an overdose of pentobarbitone. Then, they were perfused with cold 0.9% PBS, and the hearts were weighed, harvested, fixed, or frozen for histologic and molecular analyses.

### Blood Pressure Measurement

Systolic blood pressure was taken by the non-invasive tail-cuff method using BP-2000 Blood Pressure Analysis System (VisitechSystems, Apex, NC, United States). Systolic blood pressure was measured at least three times for each mouse.

### Cardiac Function Assessment by Echocardiography

A non-invasive transthoracic echocardiographic examination was performed using Vevo 2,100 (Visualsonics, Canada), equipped with a 30 MHz transducer. The mice were anesthetized with continuous flow of 1–2% isoflurane. Two-dimensional guide M-mode tracings were recorded, ejection fraction (EF), and fractional shortening (FS) were measured and further calculated.

### Histology and Immunohistochemistry

Histological analyses were performed essentially as described (Li et al., 2020). Paraffin sections were stained with hematoxylin and eosin (HE, Servicebio, China), or Masson’s trichrome (Servicebio, China) according to standard procedures. Immunofluorescence staining was performed with indicated primary antibodies overnight at 4°C and secondary antibodies conjugated with FITC or Texas Red (Thermo Fisher Scientific, United States) for 30 min at room temperature. Wheat germ agglutinin staining (WGA) was applied following the manufacturer’s instructions (Invitrogen, W11261, CA, United States). Pictures were taken using a fluorescence microscope (Axio Imager M2; Carl Zeiss, Oberkochen, Germany). Quantifications were performed with ImageJ software. Primary antibodies used in Immunohistochemistry include KLF15 (Santa Cruz Biotechnology, sc-271675, United States), ACTA2 (Abcam, ab7817, United States), F4/80 (Servicebio, GB11027, China), and *CXCR2* (Abclonal, A3301, China).

### Cell Culture, Infection, Transfection, Reporter Assay, and Elisa

CFs were isolated according to manufacturer’s instruction (MACS, 130-098-373, Germany). H9c2 cells and HEK293T cells were purchased from ATCC (CRL-1466, CRL-3216). CFs and H9c2 cells were cultured in a complete medium containing DMEM supplemented with 10% fetal bovine serum (FBS; Thermo Fisher Scientific, United States), 100 U/ml penicillin, and streptomycin. Bone marrow-derived macrophages (BMDM) were isolated by flushing mice femur and tibia with a syringe and a 26-gauge needle with RPMI1640 supplemented with 100 U/ml penicillin, 100 μg/ml streptomycin and 0.2% fetal bone serum (FBS). After centrifugation, the pellet was resuspended with RPMI1640 with 10%FBS. Cells were stimulated by 50ng/ml M-CSF for 7 days to obtain BMDM. Cells were cultured at 37°C in a humidified atmosphere containing 5% CO_2_. Transient transfections were performed with jetPRIME^®^ (Polyplus Transfection, United States) according to the manufacturer’s instructions. AdCTL, AdKLF15, or AdKLF15-ΔTAD was infected as previous described (Lu et al., 2019). Small interfering RNAs were constructed by Genepharma Co. (Shanghai, China). Cells were harvested after stimulation by vehicle or Ang II with the indicated concentration and time after 48 h of transfection. The human *CXCL1* promoter covering a region from −2,000 to +1 was ligated into a pGL4.10 luciferase reporter vector and transfected into HEK293T cells by Lipofectamine 3,000. Reporter activity was measured using a luciferase reporter assay system (Promega, E1910/E1960, United States) as previously described (Lu et al., 2019). Cell supernatant was collected for a *CXCL1* Elisa assay according to the manufacturer’s instructions (Raybiotech, ELM-KC-1, United States).

### Protein Extraction and Western Blot

Proteins were extracted from heart tissue or cells using RIPA lysis buffer (Millipore, HY-K0010) with a proteinase inhibitor cocktail (Millipore, HY-K0010). The protein was separated by 10% SDS-PAGE gel and then transferred onto PVDF membranes (Millipore, IPFL00005, United States). Membranes were blocked with 5% non-fat milk in TBST at room temperature for 60 min and incubated overnight with indicated primary antibodies at 4°C, followed incubation with HRP-linked secondary antibodies at room temperature for 60 min, and the specific proteins were detected by an ECL Detection System (Pierce, Rockford, IL, United States). The primary antibodies used in Western Blot include KLF15 (Proteintech, 66185, China), P-mTOR (CST, 5536, United States), mTOR (CST, 2983, United States), P-NF-κB p65 (CST, 3039, United States), NF-κB p65 (CST, 8242, United States), P-ERK1/2 (CST, 4370, United States), ERK1/2 (CST, 4695, United States), *CXCL1* (ABclonal, A5802, China),*CXCR2* (ABclonal, A3301, China), GAPDH (CST, 8884, United States), Flag (Sigma, F1804, United States), and anti-rabbit or anti-mouse secondary antibodies (CST, 7074 or 7076, United States).

### RNA Isolation and Real-Time PCR

Total mRNA was extracted from heart tissue or cells by the commercial RNA purification kit (EZBioscience, B0009, United States) following the manufacturer’s instruction. And 1,000 ng of total mRNA was reverse-transcribed into cDNA using HiScript III RT SuperMix (Vazyme, R323, China). Realtime PCR reactions were performed using a commercial SYBR Green kit (Vazyme, Q311-02, China) on an ABI Prism StepOne Plus system (ABI, United States). Sequences of the primers were listed in Supplementary Table S1.

### Statistical Analysis

A *t*-test for two groups and one-way ANOVA or two-way ANOVA with Tukey’s *post hoc* test for multiple groups were performed by Prism 6 (GraphPad, La Jolla, CA, United States). *P*-values less than 0.05 were considered statistically significant.

## Results

### Ang II Induced Decrease of KLF15 Expression Associated With Increase of *CXCL1/CXCR2* Expression

**FIGURE 1:**
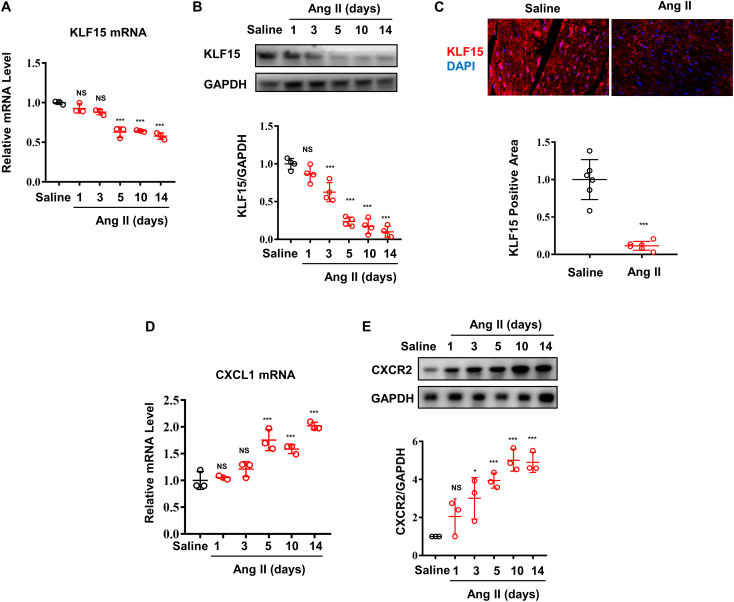
Ang II induced decrease of KLF15 expression associating with increase of *CXCL1/CXCR2* expression. Mice were infused with saline or Ang II (1,000 ng/kg/min) for the indicated time. **(A)** Cardiac KLF15 mRNA was detected and analyzed by qPCR. **(B)** KLF15 protein expression was detected and analyzed by Western Blot. **(C)** Representative immunofluorescence image of KLF15 in mice heart section (upper) and quantification analysis (lower). **(D)** *CXCL1* mRNA was measured and analyzed by qPCR. **(E)** *CXCR2* protein was detected and analyzed by Western Blot. *P < 0.05 and ***P < 0.001.

Our previous study found that Ang II induced decrease of KLF15 in adventitial fibroblasts (Lu et al., 2019). In this study, the expression of cardiac KLF15 was measured in WT mice infused with Ang II. Ang II decreased cardiac KLF15 mRNA and protein expression in a time-dependent manner (Figures 1A,B). The decrease of KLF15 was also validated by immunofluorescence (Figure 1C). It is reported that *CXCL1/CXCR2* axis mediates Ang II-induced cardiac hypertrophy and remodeling (Wang et al., 2018). In addition, inspired by the RNA-seq results that identified several KLF15-regulated chemokines expression in smooth muscle cells (Sasse et al., 2017), we found that Ang II increased cardiac *CXCL1* expression (Figure 1D), which showed an opposite trend compared with cardiac KLF15 expression. Moreover, *CXCR2*, the receptor of *CXCL1*, was also significantly increased after Ang II infusion (Figure 1E). These findings suggested that Ang II may induce *CXCL1/CXCR2*-associated inflammatory response via suppressing KLF15 expression.

### Deficiency of KLF15 Aggravated Ang II-Induced Cardiac Hypertrophy and Fibrosis

**FIGURE 2:**
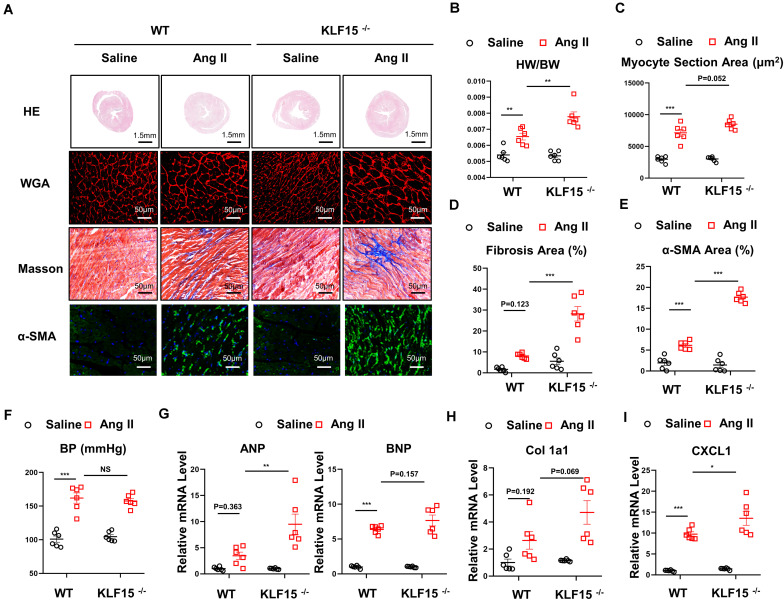
Deficiency of KLF15 aggravated Ang II-induced cardiac remodeling. Wild-type (WT) mice and KLF15 knockout (KO) mice were infused with saline or Ang II for 14 days. **(A)** Representative heart size, WGA stain, Masson stain, and α-SMA immunofluorescence image. **(B)** Statistical analysis of heart weight/body weight ratio. **(C)** Quantification analysis of myocyte section area. **(D)** Quantification analysis of fibrotic area measured by Masson stain. **(E)** Quantification analysis of α-SMA positive area measured by immunofluorescence. **(F)** Statistical analysis of blood pressure. **(G–I)** qPCR analysis of mRNA levels of ANP, BNP, Collagen 1a1, and *CXCL1.* *P < 0.05, **P < 0.01, and ***P < 0.001.

Next, WT and KLF15 KO mice were used to investigate the effect of KLF15 on Ang II-induced cardiac remodeling. After 2 weeks of Ang II treatment, the cardiomyocyte hypertrophy was markedly aggravated in the KLF15 KO mice, which was revealed by histological analysis with HE and WGA, heart weight, and body weight (Figures 2A–C). In addition, KLF15 KO aggravated Ang II-induced cardiac fibrosis revealed by Masson stain (Figures 2A,D). Accordingly, myofibroblasts showed more activated in KLF15 KO mice tested by immunofluorescence of α-SMA positive cells (Figures 2A,E). However, the blood pressure levels between KLF15 KO and WT mice were indistinguishable (Figure 2F). Moreover, mRNA levels of ANP and BNP were higher in KLF15 KO mice compared with the WT mice (Figure 2G), which indicated deteriorated cardiac hypertrophy. Collagen 1a1 mRNA level was also higher in KLF15 KO mice which revealed aggravated fibrosis (Figure 2H). Most importantly, *CXCL1* mRNA level showed significantly increase in KLF15 KO heart (Figure 2I).

### Deficiency of KLF15 Promoted Inflammatory Cell Infiltration and Multiple Signaling Activation

**FIGURE 3:**
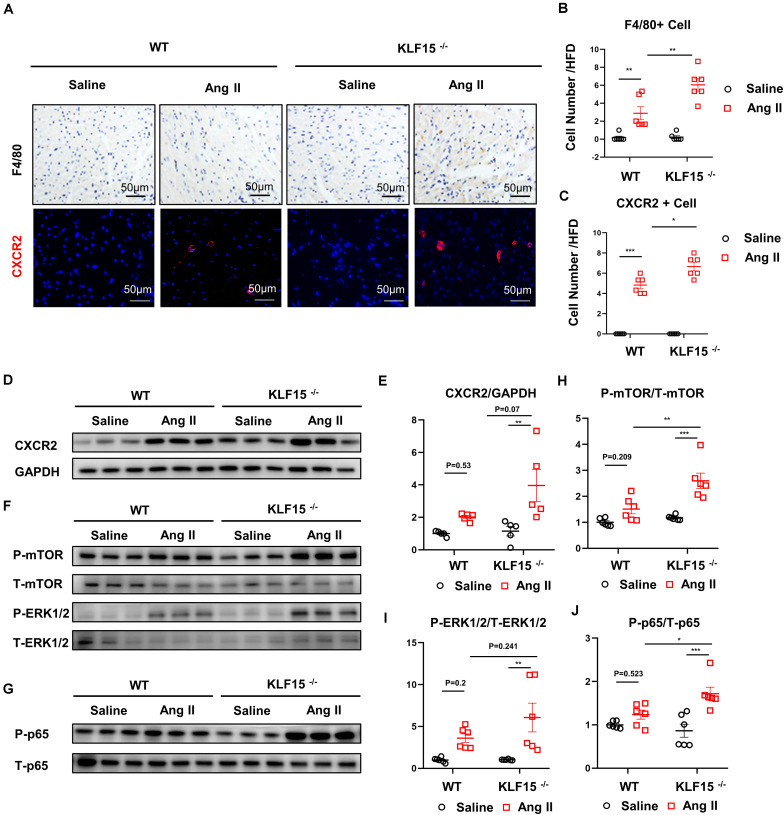
KLF15 regulated *CXCR2*-mediated inflammatory cell infiltration and downstream signal. WT and KO mice were infused with saline or Ang II for 14 days. **(A)** Representative immunohistology and immunofluorescence image of F4/80, *CXCR2* positive cell in heart. **(B,C)** Quantification analysis of F4/80, *CXCR2* positive cell infiltration in mice heart. **(D,E)** Western Blot and quantification analysis of *CXCR2*/GAPDH. **(F–J)** Western Blot and quantification analysis of P-mTOR/T-mTOR, P-ERK1/2/T-ERK1/2 and P-p65/T-p65 of mice heart. *P < 0.05, **P < 0.01, and ***P < 0.001.

*CXCL1/CXCR2* axis mediates inflammatory cell infiltration and subsequent multiple cardiac pathological signal activation (Wang et al., 2018). We found that the number of F4/80 positive and *CXCR2* positive cells were increased in the heart of KLF15 KO mice compared with WT mice (Figures 3A–C). Moreover, *CXCR2* protein level showed an increasing trend in heart of KLF15 KO mice (Figures 3D,E). To elucidate the molecular mechanism that underlying aggravated cardiac remodeling and dysfunction in KLF15 KO mice, we examined multiple signaling pathways that mediate cardiac pathological changes (Wang et al., 2018). We found that Ang II-induced increase of P-mTOR, P-ERK1/2, P-p65 protein levels, which were all remarkedly aggravated in the hearts of KLF15 KO mice compared with WT mice (Figures 3F–J).

### KLF15 Negatively Regulated *CXCL1* Transcription Through TAD in Cardiac Fibroblasts

**FIGURE 4:**
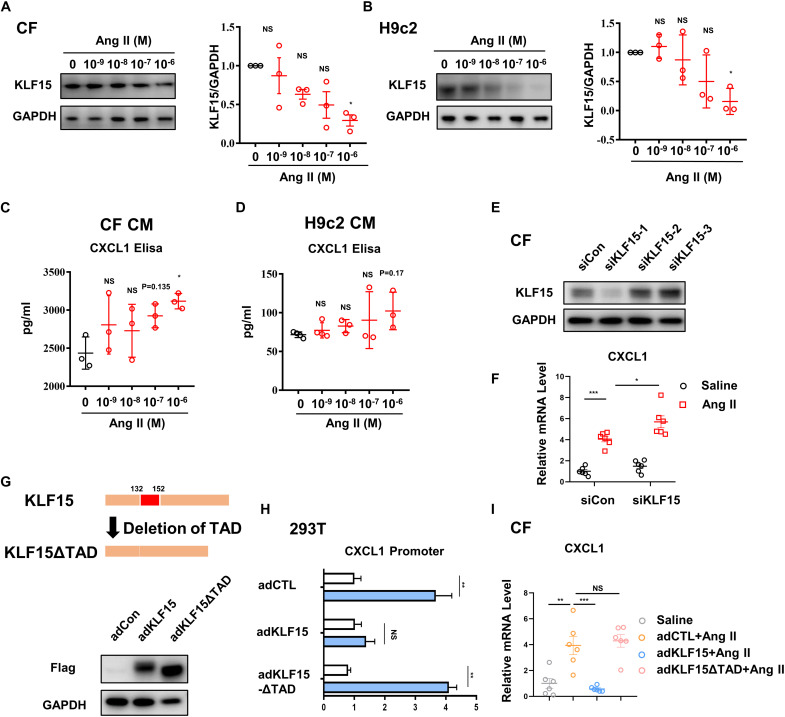
KLF15-TAD negatively regulated *CXCL1* transcription in CF. CF and H9c2 cells were stimulated by different doses of Ang II for 24 h. **(A,B)** KLF15 level was measured and quantified by Western Blot analysis. **(C,D)** The *CXCL1* protein in cell supernatant of Ang II treated CF and H9c2 cells was detected by an Elisa assay. **(E)** CF cells were transfected with con-siRNA and KLF15-siRNA. Successful knockout of KLF15 by KLF15-siRNA was confirmed by Western Blot. **(F)** *CXCL1* mRNA levels of Ang II-treated WT CF and KLF15 knockdown CF were measured by qPCR. **(G)** Adenovirus-mediated overexpression of KLF15 and deletion of transactivation domain (TAD) of KFL15 were verified by Western Blot. **(H)** Luciferase assay was used to detect *CXCL1* promoter activity in AdCTL, AdKLF15, and AdKLF15-ΔTAD-infected CFs. **(I)** *CXCL1* mRNA levels of AdCTL, AdKLF15, and AdKLF15-ΔTAD infected CFs were measured and analyzed by qPCR. *P < 0.05, **P < 0.01, and ***P < 0.001.

To determine which cell type in the heart contributes to the secretion of *CXCL1*, cardiac fibroblasts (CF) and cardiomyocytes (H9c2) were treated with different doses of Ang II. Dose-dependent decrease of KLF15 was observed both in CFs and H9c2 after Ang II stimulation (Figures 4A,B). Next, *CXCL1* expression in the supernatant of Ang II-treated CF and H9c2 cells was analyzed by ELISA assay. The results showed that Ang II induced-*CXCL1* secretion mainly derived from CF (Figures 4C,D). Western Blot was used to measure the expression of *CXCL1* and *CXCR2*. The results also showed that *CXCL1* was especially up-regulated in CFs but not H9c2 cells. Furthermore, Ang II has no effect on the expression of *CXCR2* in CFs and H9c2 cells (Supplementary Figures S1A,B). To further investigate whether KLF15 regulates *CXCL1* transcription, KLF15 expression was successful knockdown by KLF15 siRNA1 and confirmed by Western Blot (Figure 4E). SiKLF15-transfected CFs showed higher *CXCL1* expression than siCon-transfected CFs in response to Ang II (Figure 4F). Previous study has shown that KLF15 regulates inflammatory factor expression through TAD (Lu et al., 2019). We infected CFs with AdKLF15 and AdKLF15-ΔTAD to determine whether TAD is involved in KLF15-dependent *CXCL1* transcription (Figure 4G). Interestingly, luciferase and qPCR assay revealed that KLF15-overexpressed CF, but not KLF15-ΔTAD-overexpressed CF, showed a lower *CXCL1* promoter activity and *CXCL1* mRNA level (Figures 4H,I). These results indicated that KLF15 regulates *CXCL1* transcription through TAD. Furthermore, to exclude the function of macrophage, BMDM was cultured and stimulated by Ang II. We found that Ang II increased *CXCR2* expression but had no effect on the regulation of KLF15 and *CXCL1* expression in BMDM (Supplementary Figure S1C). Overexpression of KLF15 or KLF15-ΔTAD showed no effect on *CXCR2* expression in BMDM (Supplementary Figure S1D).

### KLF15 Alleviated Ang II-Induced Cardiac Hypertrophy and Fibrosis Through TAD

**FIGURE 5:**
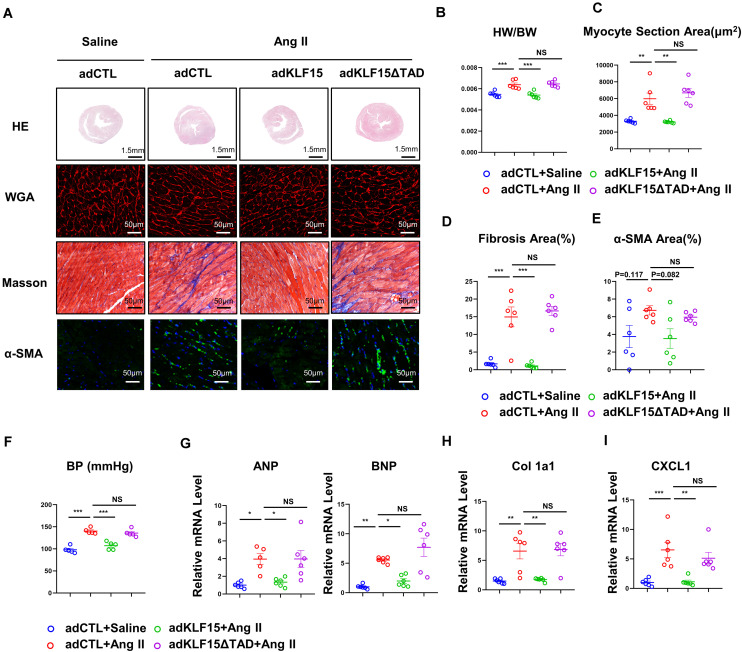
KLF15 improved Ang II-induced cardiac remodeling through TAD. AdCTL, AdKLF15 and Ad KLF15-ΔTAD-infected mice were infused with saline or Ang II for 14 days. **(A)** Representative heart size, WGA stain, Masson stain and α-SMA immunofluorescence image. **(B)** Statistical analysis of heart weight/body weight ratio. **(C)** Quantification analysis of myocyte section area. **(D)** Quantification analysis of fibrotic area measured by Masson stain. **(E)** Quantification analysis of α-SMA positive area measured by immunofluorescence. **(F)** Statistical analysis of blood pressure. **(G–I)** qPCR analysis of mRNA levels of ANP, BNP, Collagen 1a1 and *CXCL1.* *P < 0.05, **P < 0.01, and ***P < 0.001.

Next, we tested whether overexpression of KLF15 or KLF15-ΔTAD can reverse Ang II-induced cardiac hypertrophy and fibrosis. WT mice were injected with KLF15 and KLF15-ΔTAD adenovirus 1 day before Ang II infusion. After 2 weeks of Ang II infusion, AdKLF15-infected mice displayed improved cardiac hypertrophy (heart/body weight ratio, heart size, myocyte area, and mRNA level of ANP, BNP) as compared to the AdCTL-infected mice (Figures 5A–C,G). Moreover, less myocardial fibrosis, α-SMA-positive myofibroblasts, and the expression of collagen 1a1 mRNA were observed in AdKLF15 mice heart (Figures 5A,D,E,H). Interestingly, contrary to AdKLF15, AdKLF15-ΔTAD showed no effect on cardiac hypertrophy (heart/body weight ratio, heart size, myocyte area and mRNA level of ANP, BNP) compared with the AdCTL-infected mice (Figures 5A–C,G). Myocardial fibrosis, α-SMA-positive myofibroblasts, and the mRNA expression of collagen 1a1 also showed no difference between AdKLF15-ΔTAD and AdCTL-infected mice (Figures 5A,D,E,H). The improved blood pressure was observed in AdKLF15 group but not AdKLF15-ΔTAD group (Figure 5F). Consistent with the results *in vitro*, mRNA expression of *CXCL1* in heart was regulated by KLF15-TAD *in vivo* (Figure 5I). These results showed that the protective role of KLF15 in cardiac remodeling depends on its TAD.

### Inhibition of *CXCR2* Rescued KLF15 KO Aggravated Cardiac Remodeling

**FIGURE 6:**
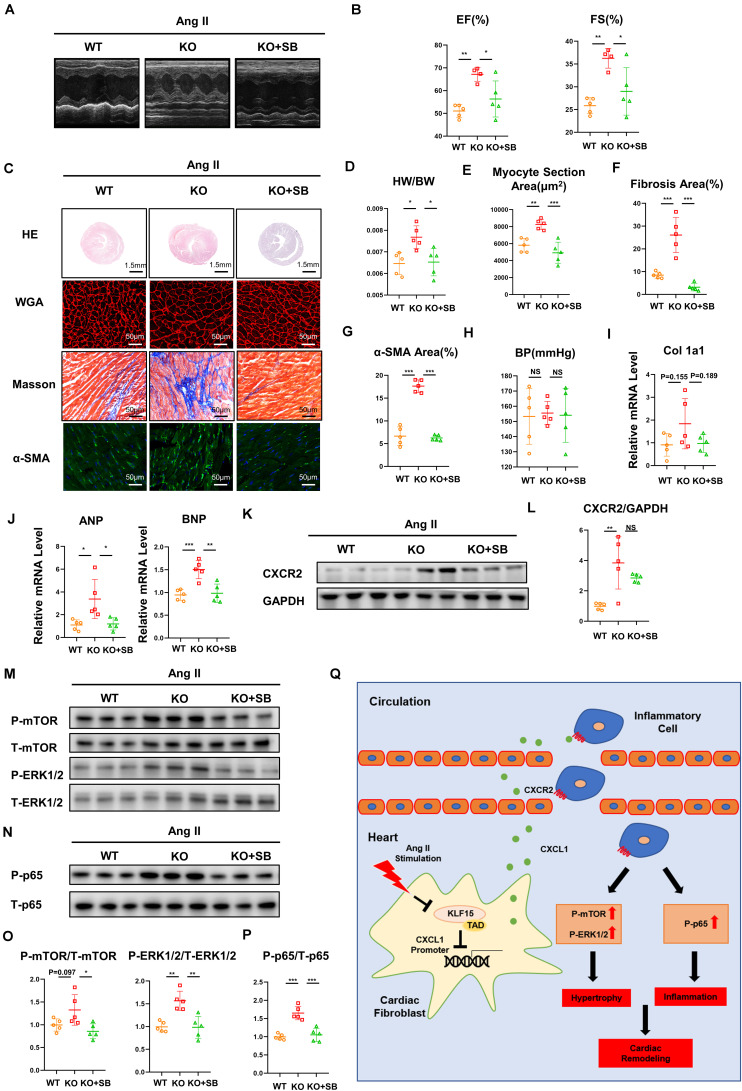
Inhibition of CXCR2 rescued KLF15 KO-aggravated cardiac remodeling. KLF15 KO mice was i.p. injected with SB265610 (2 mg/kg/day) and infused with Ang II for 14 days. WT and KLF15 KO mice only infused with Ang II were used as control. **(A)** M-mode echocardiography of left ventricular chamber. **(B)** Measurement of ejection fraction (EF%) and fractional shortening (FS%). **(C)** Representative heart size, WGA stain, Masson stain and α-SMA immunofluorescence image. **(D)** Heart weight/body weight ratio. **(E)** Quantification of myocyte section area. **(F)** Quantification of fibrotic area revealed by Masson staining. **(G)** Quantification of α-SMA positive area. **(H)** Statistical analysis of blood pressure. **(I,J)** qPCR analysis of mRNA levels of Collagen 1a1, ANP and BNP. **(K,L)** Western Blot and quantification analysis of *CXCR2*/GAPDH. **(M–P)** Western Blot and quantification analysis of P-mTOR/T-mTOR, P-ERK1/2/T-ERK1/2 and P-p65/T-p65 of mice hearts. *P < 0.05, **P < 0.01, and ***P < 0.001. **(Q)** A working model describing that KLF15 in cardiac fibroblasts negatively regulates *CXCL1/CXCR2* axis-mediated inflammatory response and subsequent cardiac remodeling in hypertension.

To confirm the effect of KLF15 on *CXCL1/CXCR2* axis *in vivo*, KLF15 KO mice was treated with a *CXCR2*-specific antagonist SB265610 (2 mg/kg, once a day) and infused with Ang II for 2 weeks. Compared with the KLF15 KO mice, both contractile dysfunction (EF and FS) and cardiac hypertrophy (heart/body weight ratio, heart size, myocyte area and mRNA level of ANP, BNP) were improved in SB265610-treated KLF15 mice (Figures 6A–E,J). Furthermore, myocardial fibrosis, α-SMA-positive myofibroblasts, and the mRNA expression of collagen 1a1 in KLF15 KO mice hearts were blunted by SB265610 treatment (Figures 6C,F,G,I). However, SB265610 showed no effect on the blood pressure (Figure 6H). The expression of *CXCR2* protein showed no significant difference between two groups, but there is a certain downward trend (Figures 6K,L). The increase of P-mTOR, P-ERK1/2, and P-p65 expression was also rescued by inhibition of *CXCR2* (Figures 6M–P).

## Discussion

Crosstalk between cardiac cells and inflammatory cells significantly contributes to cardiac remodeling (Frangogiannis, 2018). In this study, we have demonstrated that KLF15 controls cardiac inflammatory response by regulating *CXCL1* expression in the cardiac fibroblasts, which promoted *CXCR2* positive inflammatory cells infiltration and aggravated cardiac dysfunction, hypertrophy, and fibrosis. Importantly, we found that KLF15 but not KLF15-ΔTAD attenuated inflammation-associated cardiac pathological change. KLF15 negatively regulated *CXCL1* expression through TAD to mediate macrophage infiltration into injured heart. Blockade of *CXCL1/CXCR2* signaling attenuated KLF15 deficiency-induced accelerated cardiac remodeling, which provides a new KLF15/*CXCL1* axis in regulation of hypertension-associated cardiac remodeling (Figure 6Q).

A major finding is that KLF15 negatively regulates Ang II-induced cardiac remodeling through transactivation domain. Experimental evidence suggested that KLF15 functions as a transcription repressor of cardiac hypertrophy and fibrosis (Zhao et al., 2019). The KLF15 protein domain map shows highly conserved regions including the transactivation domain (Otteson et al., 2004). Functional analysis demonstrated that the KLF15 transactivation domain participates in transcription regulation of mRNA expression (Mas et al., 2011). It is reported that KLF15 TAD peptide competed with full-length KLF15 for binding to P300, and deletion of TAD also showed no effect on downstream gene expression, suggesting the functional role of KLF15 TAD in diseases (Lu et al., 2013). By using adenovirus producing full-length KLF15 or KLF15 with deletion of the transactivation domain, we found that overexpression of KLF15, but not KLF15-ΔTAD, attenuated Ang II-induced cardiac dysfunction, hypertrophy, and fibrosis. These data suggest the important role of the conservative transactivation domain in KLF15.

Chemokines play an important role in the pathogenesis of remodeling following cardiac injury by recruiting and activating inflammatory cells, which in turn exert direct effects on resident cardiac cells (Dobaczewski and Frangogiannis, 2009). Recently, we found that KLF15 negatively regulates chemokine CCL2 expression, which recruits macrophages into injured arteries in hypertension (Lu et al., 2019). In this study, we found that KLF15 negatively regulates Ang II-induced *CXCL1* expression in cardiac fibroblasts through transactivation domain. Cardiac fibroblasts traditionally recognized for their structural role in synthesizing and remodeling the ECM in tissues. Growing evidence suggests that activated cardiac fibroblasts are able to secrete different cytokines, chemokines, and growth factors to communicate with inflammatory cells (Van Linthout et al., 2014). Although KLF15 is highly expressed both in cardiac fibroblasts and cardiomyocyte (Chin, 2008), we found that Ang II induced decrease of KLF15 especially in cardiac fibroblasts. Indeed, KLF15 negatively regulates *CXCL1* expression through TAD. Knockout of KLF15 increased Ang II-induced *CXCL1* expression, while overexpression of KLF15 attenuated *CXCL1* expression. Interestingly, we further found that deletion of TAD in KLF15 failed to attenuate Ang II-induced inflammation and cardiac remodeling (Lu et al., 2019). Therefore, the protective role of KLF15 in cardiac remodeling is possible due to the decrease of *CXCL1* expression-mediated inflammatory response.

Recruitment and activation of monocytes and macrophages exert important effects on experimental model of cardiac remodeling (Burchfield et al., 2013). Recently, it has been reported that the *CXCL1/CXCR2* axis is essential for recruitment of monocytes and macrophages into hearts and arteries in hypertension (Wang et al., 2016, 2018). In keeping with those data, we found that the decrease of KLF15 expression was associated with an increase of *CXCL1* and *CXCR2* expression in heart of Ang II infused wild-type mice. Deficiency of KLF15 increased *CXCL1* expression associated with more cardiac *CXCR2*^+^ positive cells and thereby aggravated cardiac dysfunction and remodeling in response to Ang II. Furthermore, *CXCR2* inhibitors have emerged as a promising therapeutic method for the treatment of inflammatory cardiovascular disease in animal models, including ischemia/reperfusion injury, vascular injury, and cardiac remodeling (Tarzami et al., 2003; Wang et al., 2016; Wang et al., 2018). Therefore, we found that *CXCR2* inhibitors significantly attenuated KLF15 deficiency-induced exacerbation of pathological changes but not blood pressure in response to Ang II. However, a previous study found that *CXCR2* inhibitors reverse Ang II-induced elevated blood pressure (Wang et al., 2016). The inconsistence may be due to the changes of downstream factors regulated by KLF15 that counteract the antihypertensive effect of *CXCR2* inhibitors. In general, these data suggest that KLF15 in cardiac fibroblasts modulates *CXCL1/CXCR2* signaling mediated-inflammatory responses contributing to angiotensin II-induced cardiac remodeling.

Studies have shown that cardiac fibrosis and hypertrophy is involved in several activating signaling pathway including ERK1/2, mTOR, and NFκB (Zhao et al., 2015; Liu et al., 2016). A previous study showed that KLF15 suppressed isoproterenol-induced cardiac hypertrophy and fibrosis by inhibiting mTOR signaling (Gao et al., 2017). Besides, KLF15 was demonstrated to have a protective effect on atherosclerosis via inhibition of NF-κB signaling (Lu et al., 2013). Consistently with these, we found that deficiency of KLF15 promoted activation of mTOR, ERK, and NFκB signaling in hearts, thereby worsening cardiac remodeling and dysfunction. Blockade of the *CXCL1/CXCR2* axis suppressed KLF15 deficiency-mediated aggravated mTOR, ERK, and NFκB signaling pathway activation in response to Ang II, suggesting that inflammatory cells may also participate in this signaling activation.

In summary, by using both KLF15 loss- and gain-of-function mice, our results demonstrate that KLF15 through its transactivation domain modulates *CXCL1* expression-mediated pathological cardiac remodeling processes including inflammatory response, cardiac dysfunction, fibrosis and hypertrophy. Therefore, therapeutic targeting of the KLF15 or *CXCL1/CXCR2* axis may serve as an innovative approach for the treatment of hypertension-associated cardiovascular disease.

## Data Availability Statement

The raw data supporting the conclusions of this article will be made available by the authors, without undue reservation.

## Ethics Statement

The animal study was reviewed and approved by the Ethics Review Board of Ruijin Hospital, Shanghai, China.

## Author Contributions

XDL, YL, JW, and PG designed the research. SH and YL analyzed the data. SH, YL, YG, SL, XL, SS, HZ, and RW performed the research. SH, YL, XDL, and PG wrote the manuscript. All authors contributed to the article and approved the submitted version.

## Conflict of Interest

The authors declare that the research was conducted in the absence of any commercial or financial relationships that could be construed as a potential conflict of interest.

## References

[B1] Burchfield J. S. Xie M. Hill J. A. (2013). Pathological ventricular remodeling: mechanisms: part 1 of 2. *Circulation* 128 388–400. 10.1161/circulationaha.113.001878 23877061 PMC3801217

[B2] Chin M. T. (2008). KLF15 and cardiac fibrosis: the heart thickens. *J. Mol. Cell. Cardiol*. 45 165–167. 10.1016/j.yjmcc.2008.05.022 18585387 PMC2561205

[B3] Dobaczewski M. Frangogiannis N. G. (2009). Chemokines and cardiac fibrosis. *Front. Biosci*. 1 391–405. 10.2741/s33 19482709 PMC2798729

[B4] Frangogiannis N. G. (2018). Cardiac fibrosis: Cell biological mechanisms, molecular pathways and therapeutic opportunities. *Mol. Aspects Med*. 65 70–99. 10.1016/j.mam.2018.07.001 30056242

[B5] Gao L. Guo Y. Liu X. Shang D. Du Y. (2017). KLF15 protects against isoproterenol-induced cardiac hypertrophy via regulation of cell death and inhibition of Akt/mTOR signaling. *Biochem. Biophys. Res. Commun*. 487 22–27. 10.1016/j.bbrc.2017.03.087 28336438

[B6] Katz A. M. Rolett E. L. (2016). Heart failure: when form fails to follow function. *Eur. Heart J*. 37 449–454. 10.1093/eurheartj/ehv548 26497163

[B7] Kurdi M. Booz G. W. (2011). New take on the role of angiotensin II in cardiac hypertrophy and fibrosis. *Hypertension* 57 1034–1038. 10.1161/HYPERTENSIONAHA.111.172700 21502563 PMC3098302

[B8] Li X. D. Hong M. N. Chen J. Lu Y. Y. Ye M. Q. Ma Y. (2020). Adventitial fibroblast-derived vascular endothelial growth factor promotes vasa vasorum-associated neointima formation and macrophage recruitment. *Cardiovasc. Res*. 116 708–720. 10.1093/cvr/cvz159 31241138

[B9] Liu J. Zhuang T. Pi J. Chen X. Zhang Q. Li Y. (2019). Endothelial Foxp1 regulates pathological cardiac remodeling through TGF-beta1-endothelin-1 signal pathway. *Circulation* 140 665–680. 10.1161/CIRCULATIONAHA.119.039767 31177814

[B10] Liu R. van Berlo J. H. York A. J. Vagnozzi R. J. Maillet M. Molkentin J. D. (2016). DUSP8 regulates cardiac ventricular remodeling by altering ERK1/2 signaling. *Circ. Res*. 119 249–260. 10.1161/CIRCRESAHA.115.308238 27225478 PMC4938738

[B11] Lu Y. Zhang L. Liao X. Sangwung P. Prosdocimo D. A. Zhou G. (2013). Kruppel-like factor 15 is critical for vascular inflammation. *J. Clin. Invest*. 123 4232–4241. 10.1172/jci68552 23999430 PMC3785338

[B12] Lu Y. Y. Li X. D. Zhou H. D. Shao S. He S. Hong M. N. (2019). Transactivation domain of Kruppel-like factor 15 negatively regulates angiotensin II-induced adventitial inflammation and fibrosis. *FASEB J*. 33 6254–6268. 10.1096/fj.201801809R 30776250

[B13] Mas C. Lussier-Price M. Soni S. Morse T. Arseneault G. Di Lello P. (2011). Structural and functional characterization of an atypical activation domain in erythroid Kruppel-like factor (EKLF). *Proc. Natl. Acad. Sci. U.S.A*. 108 10484–10489. 10.1073/pnas.1017029108 21670263 PMC3127900

[B14] McConnell B. B. Yang V. W. (2010). Mammalian Kruppel-like factors in health and diseases. *Physiol. Rev*. 90 1337–1381. 10.1152/physrev.00058.2009 20959618 PMC2975554

[B15] McMaster W. G. Kirabo A. Madhur M. S. Harrison D. G. (2015). Inflammation, immunity, and hypertensive end-organ damage. *Circ. Res*. 116 1022–1033. 10.1161/CIRCRESAHA.116.303697 25767287 PMC4535695

[B16] Noack C. Iyer L. M. Liaw N. Y. Schoger E. Khadjeh S. Wagner E. (2019). KLF15-Wnt-dependent cardiac reprogramming up-regulates SHISA3 in the mammalian heart. *J. Am. Coll. Cardiol*. 74 1804–1819. 10.1016/j.jacc.2019.07.076 31582141

[B17] Otteson D. C. Liu Y. Lai H. Wang C. Gray S. Jain M. K. (2004). Kruppel-like factor 15, a zinc-finger transcriptional regulator, represses the rhodopsin and interphotoreceptor retinoid-binding protein promoters. *Invest. Ophthalmol. Vis. Sci*. 45 2522–2530. 10.1167/iovs.04-0072 15277472 PMC2660604

[B18] Petz A. Grandoch M. Gorski D. J. Abrams M. Piroth M. Schneckmann R. (2019). Cardiac hyaluronan synthesis is critically involved in the cardiac macrophage response and promotes healing after ischemia reperfusion injury. *Circ. Res*. 124 1433–1447. 10.1161/CIRCRESAHA.118.313285 30916618

[B19] Sasse S. K. Kadiyala V. Danhorn T. Panettieri R. A. Jr. Phang T. L. Gerber A. N. (2017). Glucocorticoid receptor ChIP-Seq identifies PLCD1 as a KLF15 target that represses airway smooth muscle hypertrophy. *Am. J. Respir. Cell Mol. Biol*. 57 226–237. 10.1165/rcmb.2016-0357OC 28375666 PMC5576583

[B20] Shirakawa K. Endo J. Kataoka M. Katsumata Y. Yoshida N. Yamamoto T. (2018). IL-10-STAT3-galectin-3 axis is essential for osteopontin-producing reparative macrophage polarization after myocardial infarction. *Circulation* 138 2021–2035. 10.1161/CIRCULATIONAHA.118.035047 29967195

[B21] Tarzami S. T. Miao W. Mani K. Lopez L. Factor S. M. Berman J. W. (2003). Opposing effects mediated by the chemokine receptor *CXCR2* on myocardial ischemia-reperfusion injury: recruitment of potentially damaging neutrophils and direct myocardial protection. *Circulation* 108 2387–2392. 10.1161/01.CIR.0000093192.72099.9A 14568904

[B22] Te Riet L. van Esch J. H. Roks A. J. van den Meiracker A. H. Danser A. H. (2015). Hypertension: renin-angiotensin-aldosterone system alterations. *Circ. Res*. 116 960–975. 10.1161/CIRCRESAHA.116.303587 25767283

[B23] Van Linthout S. Miteva K. Tschope C. (2014). Crosstalk between fibroblasts and inflammatory cells. *Cardiovasc. Res*. 102 258–269. 10.1093/cvr/cvu062 24728497

[B24] Wang L. Zhang Y. L. Lin Q. Y. Liu Y. Guan X. M. Ma X. L. (2018). CXCL1-*CXCR2* axis mediates angiotensin II-induced cardiac hypertrophy and remodelling through regulation of monocyte infiltration. *Eur. Heart J*. 39 1818–1831. 10.1093/eurheartj/ehy085 29514257

[B25] Wang L. Zhao X. C. Cui W. Ma Y. Q. Ren H. L. Zhou X. (2016). Genetic and pharmacologic inhibition of the chemokine receptor *CXCR2* prevents experimental hypertension and vascular dysfunction. *Circulation* 134 1353–1368. 10.1161/CIRCULATIONAHA.115.020754 27678262 PMC5084654

[B26] Yoda T. McNamara K. M. Miki Y. Onodera Y. Takagi K. Nakamura Y. (2015). KLF15 in breast cancer: a novel tumor suppressor? *Cell. Oncol*. 38 227–235. 10.1007/s13402-015-0226-8 25869021 PMC13004190

[B27] Zhao Q. D. Viswanadhapalli S. Williams P. Shi Q. Tan C. Yi X. (2015). NADPH oxidase 4 induces cardiac fibrosis and hypertrophy through activating Akt/mTOR and NFkappaB signaling pathways. *Circulation* 131 643–655. 10.1161/CIRCULATIONAHA.114.011079 25589557 PMC4568756

[B28] Zhao Y. Song W. Wang L. Rane M. J. Han F. Cai L. (2019). Multiple roles of KLF15 in the heart: underlying mechanisms and therapeutic implications. *J. Mol. Cell. Cardiol*. 129 193–196. 10.1016/j.yjmcc.2019.01.024 30831134

[B29] Zuo C. Li X. Huang J. Chen D. Ji K. Yang Y. (2018). Osteoglycin attenuates cardiac fibrosis by suppressing cardiac myofibroblast proliferation and migration through antagonizing LPA3/MMP2/EGFR signaling. *Cardiovasc. Res*. 114 703–712. 10.1093/cvr/cvy035 29415171

