# Supplementary material for: Krüppel-Like Factor 15 Modulates CXCL1/CXCR2 Signaling-Mediated Inflammatory Response Contributing to Angiotensin II-Induced Cardiac Remodeling
Source: Front Cell Dev Biol. 2021 Apr 1;9:644954. doi: 10.3389/fcell.2021.644954 (PMC8047332; doi:10.3389/fcell.2021.644954)
Supplement: Supplementary file 1 [file Presentation_1.PPTX]

## Slide 1
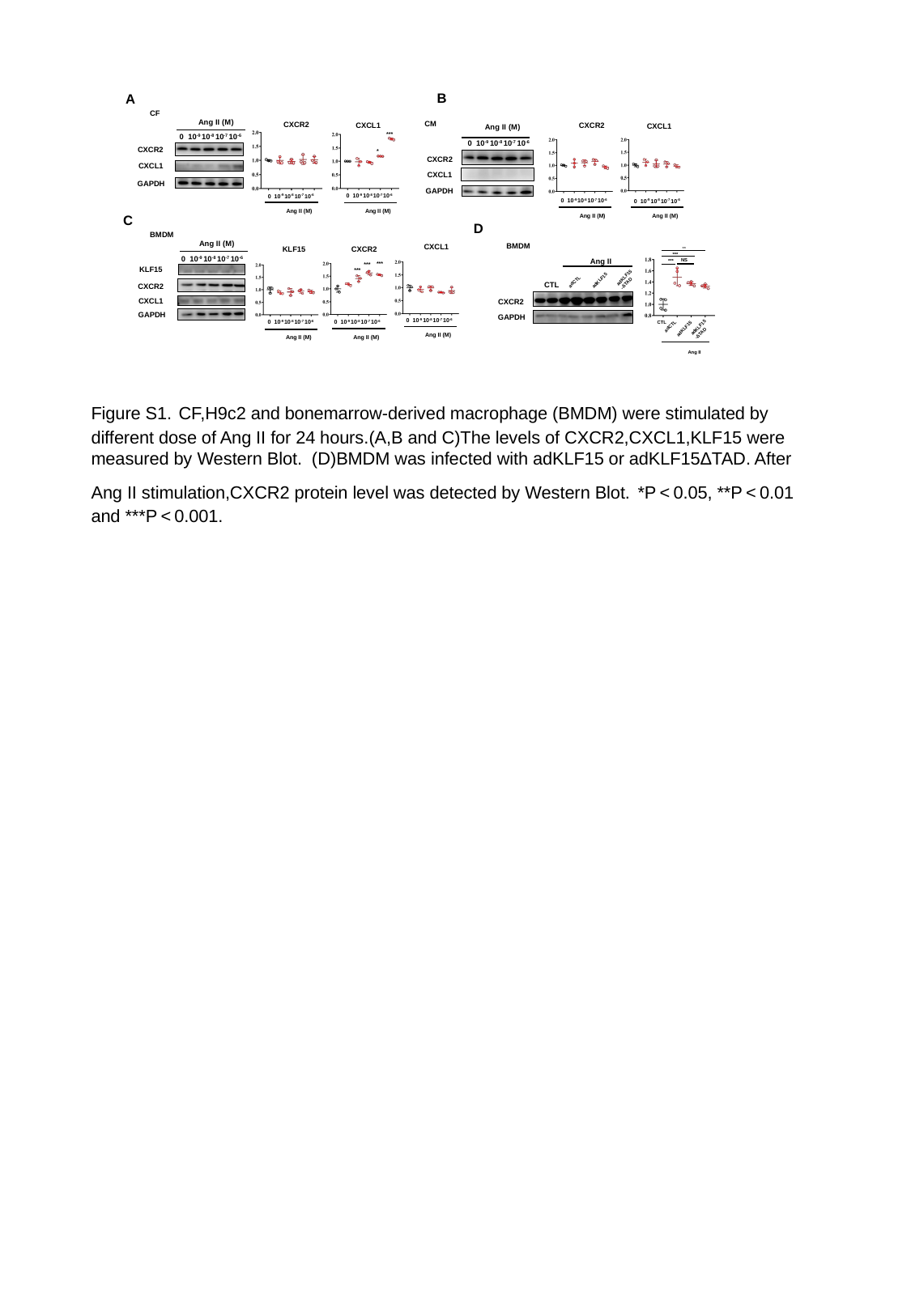

B
A
CF
Ang II (M)
CXCR2
GAPDH
CM
CXCR2
CXCL1
CXCR2
CXCL1
Ang II (M)
CXCR2
GAPDH
0 10-9 10-8 10-7 10-6
CXCL1
***
0 10-9 10-8 10-7 10-6
*
CXCL1
0 10-9 10-8 10-7 10-6
0 10-9 10-8 10-7 10-6
0 10-9 10-8 10-7 10-6
0 10-9 10-8 10-7 10-6
Ang II (M)
Ang II (M)
C
Ang II (M)
Ang II (M)
D
BMDM
Ang II (M)
CXCR2
GAPDH
0 10-9 10-8 10-7 10-6
CXCL1
BMDM
CXCL1
KLF15
CXCR2
**
***
Ang II
NS
***
***
***
KLF15
***
adKLF15
-ΔTAD
adKLF15
adCTL
CTL
CXCR2
GAPDH
0 10-9 10-8 10-7 10-6
0 10-9 10-8 10-7 10-6
0 10-9 10-8 10-7 10-6
CTL
adCTL
adKLF15
-ΔTAD
adKLF15
Ang II (M)
Ang II (M)
Ang II (M)
Ang II
Figure S1. CF,H9c2 and bonemarrow-derived macrophage (BMDM) were stimulated by different dose of Ang II for 24 hours.(A,B and C)The levels of CXCR2,CXCL1,KLF15 were measured by Western Blot. (D)BMDM was infected with adKLF15 or adKLF15ΔTAD. After Ang II stimulation,CXCR2 protein level was detected by Western Blot. *P < 0.05, **P < 0.01 and ***P < 0.001.
