# Supplementary material for: Krüppel-Like Factor 15 Modulates CXCL1/CXCR2 Signaling-Mediated Inflammatory Response Contributing to Angiotensin II-Induced Cardiac Remodeling
Source: Front Cell Dev Biol. 2021 Apr 1;9:644954. doi: 10.3389/fcell.2021.644954 (PMC8047332; doi:10.3389/fcell.2021.644954)
Supplement: Supplementary file 2 [file Table_1.DOCX]

Supplementary Material

# Supplementary Table

| **Primer** | **Sequence (5’ to 3’)** | **Species** |
| --- | --- | --- |
| KLF15 F | CAGAGAGCGTCAAGGTCGC | Mouse |
| KLF15 R | TTCGCACAAACTTTGAGGGCA | Mouse |
| Col 1a1 F | GCTCCTCTTAGGGGCCACT | Mouse |
| Col 1a1 R | CCACGTCTCACCATTGGGG | Mouse |
| ANP F | GCTTCCAGGCCATATTGGAG | Mouse |
| ANP R | GGGGGCATGACCTCATCTT | Mouse |
| BNP F | GAGGTCACTCCTATCCTCTGG | Mouse |
| BNP R | GCCATTTCCTCCGACTTTTCTC | Mouse |
| CXCL1 F | CTGGGATTCACCTCAAGAACATC | Mouse |
| CXCL1 R | CAGGGTCAAGGCAAGCCTC | Mouse |
| ACTB F | CTAAGGCCAACCGTGAAAAGAT | Mouse |
| ACTB R | GGGACAGCACAGCCTGGAT | Mouse |

KLF15, Krüppel-like factor 15; Col 1a1, Collagen type I α 1; ANP, atrial natriuretic peptide; BNP, Brain Natriuretic Peptide; CXCL1, chemokine (C-X-C motif) ligand 1; ACTB, Beta-actin.
